# Characterization of broadly neutralizing antibody responses to HIV-1 in a cohort of long term non-progressors

**DOI:** 10.1371/journal.pone.0193773

**Published:** 2018-03-20

**Authors:** Nuria González, Krisha McKee, Rebecca M. Lynch, Ivelin S. Georgiev, Laura Jimenez, Eulalia Grau, Eloísa Yuste, Peter D. Kwong, John R. Mascola, José Alcamí

**Affiliations:** 1 AIDS Immunopathology Unit, Instituto de Salud Carlos III, Madrid, Spain; 2 Vaccine Research Center, National Institute of Allergy and Infectious Diseases, NIH, Bethesda, Washington, United States of America; 3 IrsiCaixa Foundation, Hospital Universitari Germans Trias i Pujol, Universitat Autònoma de Barcelona, Badalona, Barcelona, Spain; 4 Retrovirology and Viral Immunopathology Laboratory, IDIBAPS, Hospital Clínic, University of Barcelona, Barcelona, Spain; Instituut voor Tropische Geneeskunde, BELGIUM

## Abstract

**Background:**

Only a small fraction of HIV-1-infected patients develop broadly neutralizing antibodies (bNAbs), a process generally associated to chronic antigen stimulation. It has been described that rare aviremic HIV-1-infected patients can generate bNAbs but this issue remains controversial. To address this matter we have assessed bNAb responses in a large cohort of long-term non-progressors (LTNPs) with low or undetectable viremia.

**Methods:**

Samples from the LTNP cohort of the Spanish AIDS Research Network (87 elite and 42 viremic controllers) and a control population of 176 viremic typical-progressors (TPs) were screened for bNAbs using Env-recombinant viruses. bNAb specificities were studied by ELISA using mutated gp120, neutralization assays with mutated viruses, and peptide competition. Epitope specificities were also elucidated from the serum pattern of neutralization against a panel of diverse HIV-1 isolates.

**Results:**

Broadly neutralizing sera were found among 9.3% LTNPs, both elite (7%) and viremic controllers (14%). Within the broadly neutralizing sera, CD4 binding site antibodies were detected by ELISA in 4/12 LTNPs (33%), and 16/33 of TPs (48%). Anti-MPER antibodies were detected in 6/12 LTNPs (50%) and 14/33 TPs (42%) whereas glycan-dependent HIV-1 bNAbs were more frequent in LTNPs (11/12, 92%) as compared to TPs (12/33, 36%). A good concordance between standard serum mapping and neutralization-based mapping was observed.

**Conclusion:**

LTNPs, both viremic and elite controllers, showed broad humoral immune responses against HIV-1, including activity against many major epitopes involved in bNAbs-mediated protection.

## Introduction

Production of broadly neutralizing antibodies (bNAbs) against HIV represents a relatively infrequent event in HIV-infected patients [1,2]. One major issue to induce such antibodies resides in the high variability of the viral envelope and structural mechanisms hiding crucial epitopes for neutralization. Besides, maturation leading to high affinity antibodies represents a major challenge for the immune system that can be impaired by the immunodeficiency associated with HIV infection. Affinity maturation of antibodies is critical to confer effective neutralization against HIV and this maturation capacity becomes altered along infection [3–5].

Despite the complexity of such mechanisms of viral escape, some antibodies are able to overcome these barriers and display a broad neutralizing activity. These bNAbs are mainly directed to four vulnerable Env regions: the gp120 CD4-binding site (CD4bs) [6–10], the gp41 membrane proximal external region (MPER) [11–13], glycan-dependent epitopes in the second hypervariable loop (V2) [14–16] and glycan dependent epitopes around the third hypervariable loop (V3) [11,15]. In addition these four well-established sites, new epitopes at the gp120-gp41 interface recognized by some more recently discovered bNAbs have been identified [17–20].

The study of the mechanism of action of bNAbs is essential to understand the mechanisms of antibody neutralization and escape by HIV-1. Several studies have suggested that the development of neutralizing antibodies is a consequence of viral replication [1,21]. On the other hand, it is generally accepted that bNAbs are not able to contribute the control of viremia due to continuous escape by HIV from immune pressure through mutation or glycosylation. However, it has been recently described a role of bNAbs in HIV control in one patient with EC phenotype raising the possibility of an active role of bNAbs in the control of autologous viruses [22].

We have shown that patients receiving antiretroviral treatment are capable of inducing a broad and potent humoral immune response against HIV despite having undetectable levels of viremia [23]. According to these results, it is possible that long-term nonprogressors (LTNPs), individuals with low levels of viremia who maintain stable CD4 T cell counts over 10 years of infection, develop neutralizing antibodies with a high affinity profile. In fact, in isolated LTNP patients the presence of bNAbs has been described [24–26]. We have explored the hypothesis that preserved B cell function in LTNPs could result in the production of a broad humoral response. To get a better understanding of this issue, we have assessed the presence of bNAbs in a large cohort of LTNP, including both viremic and elite controllers. Furthermore we have characterized the epitopes targeted by bNAbs found in LTNPs in comparison with those in HIV typical progressors (TPs).

## Material and methods

### HIV-1 infected subjects

This study has been approved by Research Ethics and Animal Welfare Committee of Instituto de Salud Carlos III (CEI PI 42_2011-v2).

Samples (129) from the cohort of LTNPs from the RIS (median RNA copies/ml: 104, median CD4+: 734 cells/μl and asymptomatic HIV infection over 10 year after seroconversion) were kindly provided by the HIV BioBank integrated in the Spanish AIDS Research Network (RIS) [27]. The HIV BioBank, integrated in the Spanish AIDS Research Network, is partially funded by the RD12/0017/0037 project as part of the Plan Nacional R + D + I and cofinanced by ISCIII- Subdirección General de Evaluación y el Fondo Europeo de Desarrollo Regional (FEDER) and Fundación para la investigación y prevención del SIDA en España (FIPSE). Samples were processed following current procedures and frozen immediately after their reception. All patients participating in the study gave their informed consent and protocols were approved by institutional ethical committees. A population of 176 untreated TPs (median RNA copies/ml: 10,241, median CD4+: 567 cells/μl) from Hospital Clinic, Barcelona, was analyzed as control [23]. The overall rate of CD4 cell decline in TPs was 50–100 cells/μL per year. Patients in the present study signed informed consent. All subjects on this study were antiretroviral naïve at the time of sampling.

LTNPs were classified as elite and viremic controllers (Table 1). Among LTNPs, 87 of them were elite controllers with persistent viral load below 50 RNA copies/ml and a median CD4^+^ T cell count of 773 cells/μl and 42 were viremic controllers. Viremic controllers had a median number of viral RNA copies/ml in plasma of 3,450, a median CD4^+^ T cell count of 655 cells/μl and viral load was always below 10000 RNA copies/ml.

**Table 1:** Clinical data and neutralization screening results for the patient groups.

	LTNPs		Typical progressors
	Elite controllers	Viremic controllers	
No. of sera	87	42	176
Viral RNA copies/ml plasma (median)	<50*	3,450	10,241
No. of CD4+ T cells/μl (median)	773	655	567
Years since diagnosis (median/range)	17.3 (10–25)	14.8 (10–26)	5 (0–24)
Broadly neutralizing sera	**6/87 (7%)**	**6/42 (14%)**	**33/176 (19%)**

* In some old samples (n = 34) the threshold of detection was <500 RNA copies/ml

### HIV-1 neutralization assays

To evaluate neutralizing antibody titers against HIV-1, Env-reporter viruses carrying a Renilla luciferase gene in *nef* were generated by cloning the full-length envelope in the pNL–lacZ/env–Ren vector, as previously described [28]. VI191 (A), NL4-3 (B), 92BR025 (C), 92UG024 (D), CM244 (AE) and NP1525 (CRF01_AE) envelopes were amplified from culture supernatants kindly provided by Dr. H. Holmes (NIBSC, UK) through the NeutNet consortium (Dr. G. Scarlatti) [29]. Envelopes from strains X-845-4 (F1), X-1628-2 (G), P-1261 (CRF02_AG) and 2105 (CRF14BG/B) were amplified from culture supernatants kindly provided by Dr. Lucía Pérez Álvarez, Instituto de Salud Carlos III, Spain [30]. Samples for the amplification of subtype B chronic and acute envelopes (14382, 37343, 325 and 29) were provided by Dr. José María Miró from Hospital Clinic, Barcelona. The viruses chosen represent different HIV-1 subtypes, varying neutralization sensitivity and coreceptor usage (Table 2). An amphotropic vesicular stomatitis virus (VSV) Env pseudotyped HIV-1 was added to the panel as a specificity control virus in neutralization testing.

**Table 2:** Characteristics of the viruses used in the neutralization assays.

SUBTYPE	VIRUS CODE	CORECEPTOR USAGE	INFECTION STAGE	TIER
A	VI191*	R5	Chronic	2
B	NL4-3*	X4	Chronic	1A
B	14382	R5	Acute	n.d.
B	37313	R5	Acute	n.d.
B	325	X4	Chronic	n.d.
B	29	R5X4	Chronic	n.d.
C	92BR025*	R5	Chronic	1B
D	92UG024	X4	Chronic	2
AE	CM244*	R5	Chronic	2
F1	845_4	R5	Chronic	n.d.
G	1628_2	R5	Chronic	n.d.
CRF02_AG	1261	R5X4	Chronic	n.d.
CRF14_BG/B	2105	R5	Chronic	n.d.
CRF01_AE	NP1525	X4	Chronic	n.d.

* Recombinant viruses included in the mini-panel

Infectious supernatants were generated by calcium phosphate transfection in HEK 293 T cells with 5 μg of the plasmids [31]. Titrated recombinant viruses were preincubated with the dilutions of sera (1/200-1/2000) for 30 minutes at 37°C before the infection of the U87.CD4.CCR5 or U87.CD4.CXCR4 cells (2x10^4^ per well) [32]. Virus infectivity was determined 48 h postinoculation by measuring luciferase activity in cell lysates using a 96-well plate luminometer (Orion, Berthold). Sigmoid curves were generated and ID50 neutralization titers were calculated by non-linear regression using GraphPad Prism version 7.02 software. In a first screening, serum samples were tested with a minipanel of four recombinant viruses with envelopes from different subtypes and tropisms and a VSV-pseudotyped virus. Selected serum samples neutralizing all the viruses in the minipanel with an ID50≥200 were screened against a panel of 10 more viruses.

### Epitope mapping

#### i. Neutralization assays

For the characterization of neutralizing antibodies, neutralization assays were performed using single-round infection HIV-1 Env pseudoviruses and TZM-bl target cells as previously described [33,34]. To determine the serum concentration producing 50% reduction in RLU value, serial dilutions were made and the neutralization dose-response curves were fitted by non-linear regression using a four-parameter hill slope equation.

For the assessment of CD4bs-directed neutralization, antibody-mediated neutralization was blocked with specific protein probes in a competition assay [7]. Briefly, 25 μg/ml of RSC3 (Resurfaced Stabilized Core 3) or 25 μg/ml of the mutant RSC3 Δ371I/P363N was incubated with sera serially diluted 4-fold starting from 1:10 for 30 min. JR-FL (subtype B), RW020 (subtype A) or ZA012 (subtype C) pseudovirus was added 30 min before the addition of TZM-bl cells and the infection proceeded for 48 h. CD4bs-directed activity was calculated as a 30% reduction in the ID50 values of the sera in the presence of RSC3 compared to RSC3 Δ371I/P363N [35].

For the mapping of bNAbs directed to glycan structures in the variable region (V1V2 and V3), neutralization assays in TZM-bl were performed using JR-CSF virus with the N160K mutation and the N332A mutation respectively [36,37]. The mutation of N160 to lysine, an N-glycosylation site in the V2 loop, abolishes the neutralization mediated by PG9 and PG16 and N332A mutation removes a glycosylation site at the base of V3 loop essential for the formation of epitopes recognized by 2G12 and PGT bNAbs. A sample is considered positive if there is a decrease in ID50 greater than or equal to 50% for the mutant compared to the wild-type virus [38].

For the mapping of bNAbs to the membrane-proximal external region (MPER) of gp41, sera were tested for neutralizing activity against a chimeric HIV-2 virus containing the HIV-1 MPER region of gp41 (71312-C1) and the parenteral HIV-2 7312A clone [39].

The serum neutralizing antibodies were also mapped to the MPER region by a method of selective peptide inhibition of neutralization [40]. gp41-specific overlapping peptides (MPR.03: KKKNEQELLELDKWASLWNWFDITNWLWYIRKKK, 2F5.01: NEQELLELDKWASLWN, 4E10.22: CNWFDITNWLWYIRKKK and Z13e1.01: WASLWNWFDITNKKKK) were added to the serum for 30 min at a concentration of 25 μg/ml prior to the addition of 7312A-C1 virus (HIV-2/YU2 MPER chimera). A scrambled MPER region peptide (MPR.Scr.02: KKKRIYWLWNTIDFWNWLSAWKDLELLEQENKKK) was included as negative control. In these assays the reduction of the neutralization activity caused by MPER derived peptides is compared to that due to the mock peptide (defined as an equivalent volume of DMEM medium).

#### ii. ELISA analyses

ELISA assays were performed as previously described [7]. Plates were coated with the antigen in PBS at 2 μg/ml and incubated overnight at 4°C. The ELISA plates were coated with the following probes:

- YU2 gp120wt and YU2 gp120 D368R protein. The mutation at position 368 reduces or knocks out binding of most CD4bs Ab.
- The antigenically resurfaced glycoprotein RSC3 containing the CD4bs and RSC3 Δ371I/P363N mutant, which affects the CD4 binding loop and reduces b12 and VRC01 binding.
- The RSC3 G367R probe [7,35] that creates a steric clash for mAb b12 binding but it causes little interference with VRC01 binding.

Sera that showed a loss of reactivity to the CD4bs mutants (YU2 D368R and RSC3 Δ371I/P363N) and reacted to YU2, RSC3 and RSC3 G367R were classified as containing CD4bs antibodies as described previously [35,41]. In those samples with low reactivity to YU2 gp120wt or/and RSC3 (endpoint titer below or equal to 2500) ELISA with RSC3 G367R probe was not performed.

#### iii. Neutralization-based serum delineation analysis

Serum specificities were delineated using a neutralization fingerprint algorithm, as described previously [42]. Briefly, a reference set of monoclonal antibody-virus neutralization data was obtained for a set of 21 diverse HIV-1 strains (subtypes A, B and C) against a set of representative monoclonal antibodies, divided into epitope-specific antibody clusters (VRC01, b12, CD4, HJ16, 8ANC195, PG9, PGT128, 2G12, 2F5 and 10E8-like). For each serum, the pattern of neutralization of the same set of 21 strains was compared to the neutralization patterns (fingerprints) of the set of reference antibodies, and the relative contribution of antibodies from each cluster to the neutralization by a given serum was estimated.

## Results

In order to better understand the breadth and the spectrum of neutralization against HIV-1 in LTNP patients, we evaluated the neutralizing capacity of sera samples from the cohort of LTNPs of the Spanish AIDS Research Network (RIS). The neutralizing activity in LTNPs was compared with that in the control group of TPs. In these neutralization assays, neutralizing activity of these sera were tested against a mini-panel of recombinant viruses with different subtypes (clades and tier categorizations are given in parentheses): VI191 (A, tier 2), NL4-3 (B, tier 1A), 92BR025 (C, tier 1B) and CM244 (AE, tier 2) (S1 Fig) [10,43–45]. A VSV-pseudotyped HIV-1 was included as a control for nonspecific neutralizing activity and none of the sera showed neutralizing activity against VSV-pseudotyped HIV-1. We considered that a serum sample displayed broadly neutralizing activity (bNA) when it was capable of neutralizing all the recombinant viruses tested across with a titre ≥200, with no neutralization of the VSV-pseudotyped control.

**Fig 1:**
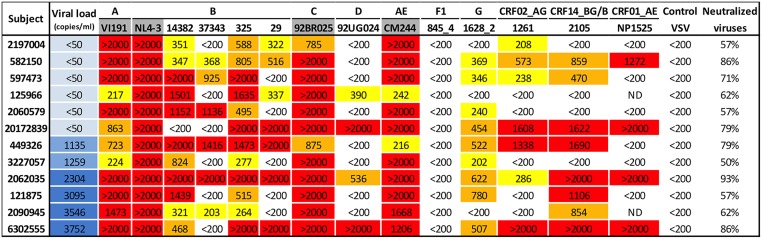
Serum neutralization data (ID50s) for LTNPs capable of neutralizing all the viruses from the mini-panel. Selected sera samples from the LTNP cohort capable to neutralize (ID50≥200) the mini-panel of 4 viruses (NL4-3, VI191, 92BR025 and CM244) were screened for neutralization against 10 more viruses. Viruses of the minipanel are shaded grey. Median viral loads for the previous five years to the serum sample are shown. In the last column the percentage of neutralized viruses is shown. No significant neutralization of the VSV pseudotyped virus was observed in any of the samples. Reciprocal serum ID50 values ≥200 and <400 are highlighted in yellow, ≥400 and <1000 in orange and ≥1000 in red.

LTNP serum samples capable to neutralize all the viruses in the mini-panel with an ID50≥200 were selected and these samples were screened against an extended panel of 10 viruses more with various subtypes (Fig 1).

The percentage of individuals with broad neutralizing responses found among LTNPs was 9.3% (12/129) and in TPs was 18.8% (33/176).

Although the number of broad neutralizer individuals in the viremic population was higher, broad neutralizer patients were also found among the elite controllers. Six of the LTNPs with bNA had viral loads under 50 copies/ml indicating that patients with undetectable viral loads are capable to develop a broadly neutralizing response. These six individuals represent 7% of the elite controllers´ population (6/87) while in the case of the viremic LTNPs the ratio of individuals with bNA was 14% (6/42) (Table 1).

The epitopes for the binding of the neutralizing antibodies contained in LTNPs sera and in TPs sera from patients with a broad neutralization profile were mapped.

NAbs directed to the CD4bs were identified with ELISA techniques. A serum was considered positive if there was a 5-fold or greater difference in serum binding to the wild type compared to the corresponding CD4bs knockout mutant probe and if it showed a good reactivity to YU2 gp120wt or/and RSC3 (endpoint titer above 2500). Sera with NAbs to the CD4bs were detected in several samples of LTNPs (597473, 3227057, 2090945 and 449326) and also in TPs (670–002, 521–006, 282–046, 363–014, 651–003, 308–040, 661–002, 328–017, 380–017, 530–013, 390–012, 706–000, 738–000, 97–031, 53–036, 642–007) (Table 3).

**Table 3:** Serum endpoint titers in ELISAs to determine the presence of CD4bs antibodies.

Subject	YU2 core	YU2 D368R	Ratio^a^	RSC3	RSC3 Δ371I/P363N	Ratio^a^	RSC3 G367R	RSC3 Δ371I/P363N	Ratio^a^
**LTNPs**									
**ECs**									
2197004	62500	2500	25	2500	2500	1	2500	2500	1
582150	2500	2500	1	2500	100	25	nd	100	nd
**597473**	312500	12500	**25**	312500	500	**625**	1562500	500	**3125**
125966	312500	62500	5	12500	2500	5	2500	2500	1
2060579	62500	12500	5	100	100	1	nd	100	nd
20172839	12500	2500	5	2500	100	25	100	100	1
**VCs**									
**3227057**	312500	12500	**25**	62500	500	**125**	2500	500	**5**
2062035	1562500	62500	25	312500	12500	25	12500	12500	1
**2090945**	62500	12500	**5**	12500	500	**25**	2500	500	**5**
**449326**	312500	12500	**25**	1562500	12500	**125**	62500	12500	**5**
121875	62500	12500	5	62500	12500	5	12500	12500	1
6302555	62500	12500	5	100	500	0,2	nd	500	nd
**TPs**									
359–016	nd	nd		2500	100	25	100	100	1
734–000	62500	12500	5	2500	2500	1	nd	2500	nd
**670–002**	nd	nd		12500	2500	**5**	12500	2500	**5**
**521–006**	312500	12500	**25**	62500	2500	**25**	12500	2500	**5**
600–003	312500	312500	1	312500	62500	5	312500	62500	5
344–017	nd	nd		2500	500	5	2500	500	5
72–071	nd	nd		2500	100	25	2500	2500	1
339–017	nd	nd		2500	2500	1	2500	2500	1
269–049	nd	nd		12500	500	25	500	500	1
378–017	nd	nd		500	100	5	500	100	5
**282–046**	nd	nd		12500	100	**125**	12500	100	**125**
322–012	nd	nd		12500	500	25	500	500	1
**363–014**	312500	62500	**5**	12500	2500	**5**	62500	2500	**25**
**651–003**	nd	nd		12500	500	**25**	2500	500	**5**
701–000	nd	nd		2500	2500	1	2500	2500	1
**308–040**	312500	12500	**25**	1562500	500	**3125**	2500	500	**5**
**661–002**	nd	nd		12500	100	**125**	500	100	**5**
56–024	nd	nd		2500	500	5	500	500	1
139–020	nd	nd		500	100	5	100	100	1
**328–017**	nd	nd		2500	500	**5**	2500	500	**5**
**380–017**	nd	nd		12500	2500	**5**	12500	2500	**5**
629–005	nd	nd		500	500	1	500	500	1
**530–013**	nd	nd		312500	500	**625**	12500	500	**25**
**390–012**	nd	nd		12500	500	**25**	2500	500	**5**
**706–000**	nd	nd		62500	100	**625**	12500	100	**125**
528–006	312500	62500	5	62500	2500	25	2500	2500	1
622–005	nd	nd		500	100	5	500	100	5
**738–000**	nd	nd		12500	500	**25**	12500	500	**25**
**97–031**	nd	nd		62500	12500	**5**	62500	12500	**5**
708–002	nd	nd		2500	100	25	500	100	5
376–036	nd	nd		2500	500	5	2500	500	5
**53–036**	nd	nd		12500	500	**25**	2500	500	**5**
**642–007**	312500	2500	**125**	1562500	2500	**625**	62500	2500	**25**

nd: not determined; ECs: Elite controllers; VCs: Viremic controllers; TPs: Typical progressors

^a^Ratio of previous two endpoint titers. Sera with a ratio RSC3 Δ371I/P363N and a loss of activity on the cognate CD4bs mutant greater than or equal to 5-fold are considered reactive and bolded.

**Fig 2:**
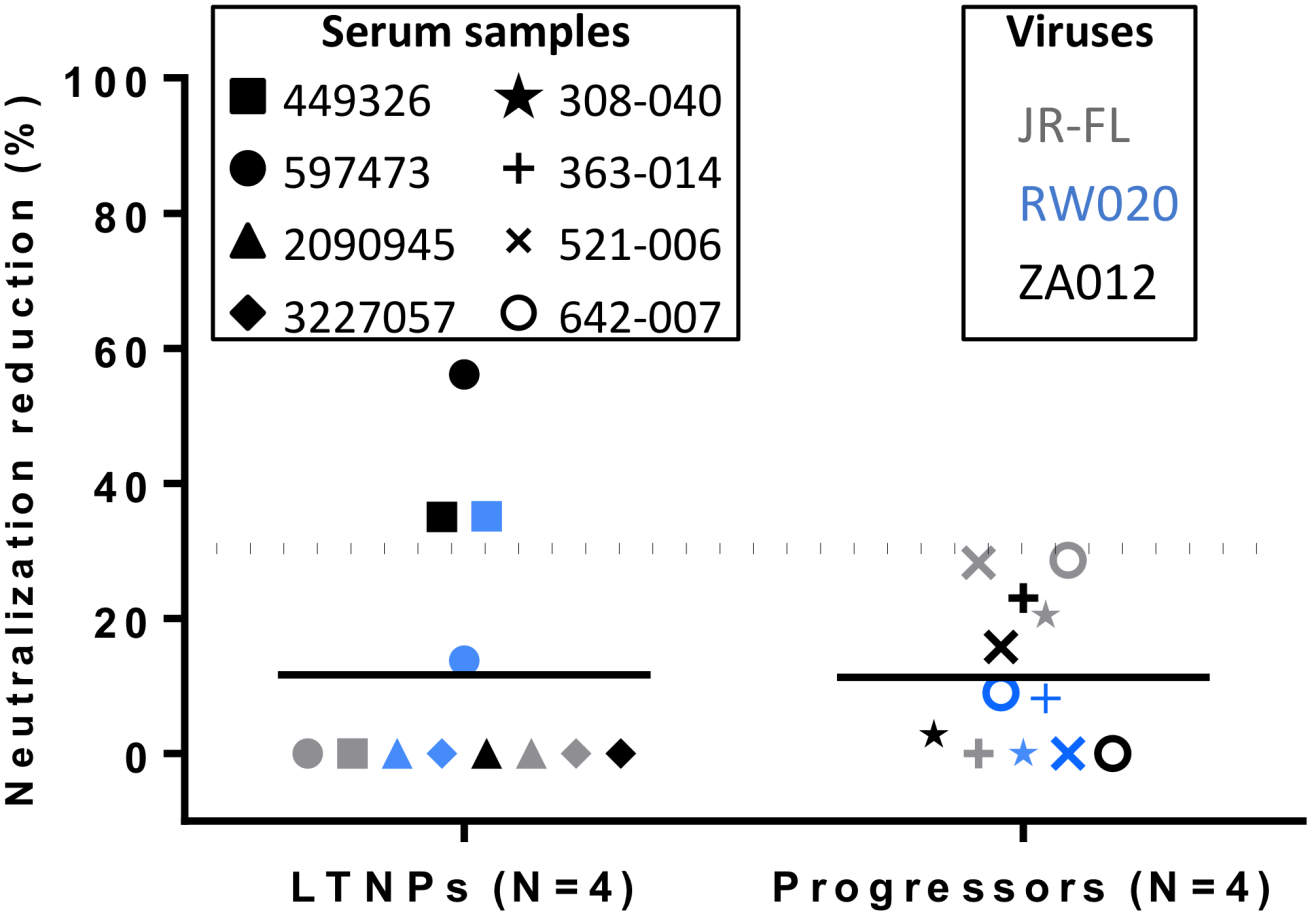
CD4 binding site neutralizing antibody specificity. Sera that were ELISA reactive with resurfaced stabilized cores RSC3 and RSC3 G367R, were assayed for inhibition of neutralization using RSC3 and RSC3 P363N as inhibitors. The net percent reduction in neutralization of JRFL, RW020 or ZA012 attributed to RSC3 compared to RSC3 P363N is plotted (y axis). Each serum is represented by a different symbol. The color of the symbol depends on the virus used in the neutralization assay. A reduction greater than 30% is considered positive. RSC3 addition inhibited neutralization mediated by 2 sera from LTNPs (449326 and 597473) and no serum of typical progressors.

To determine whether the CD4bs antibodies contained in sera were responsible for broad neutralization, neutralization competition assays using RSC3 glycoprotein containing the CD4bs were performed (Fig 2). RSC3 addition inhibited neutralization mediated by two samples of sera from LTNPs (449326 and 597473). For sample 449326 RSC3 addition inhibited neutralization of RW020 (35.2%) and ZA012 (35.1%). For serum 597473 there was a 56% reduction in neutralization of ZA012 strain attributed to RSC3.

**Fig 3:**
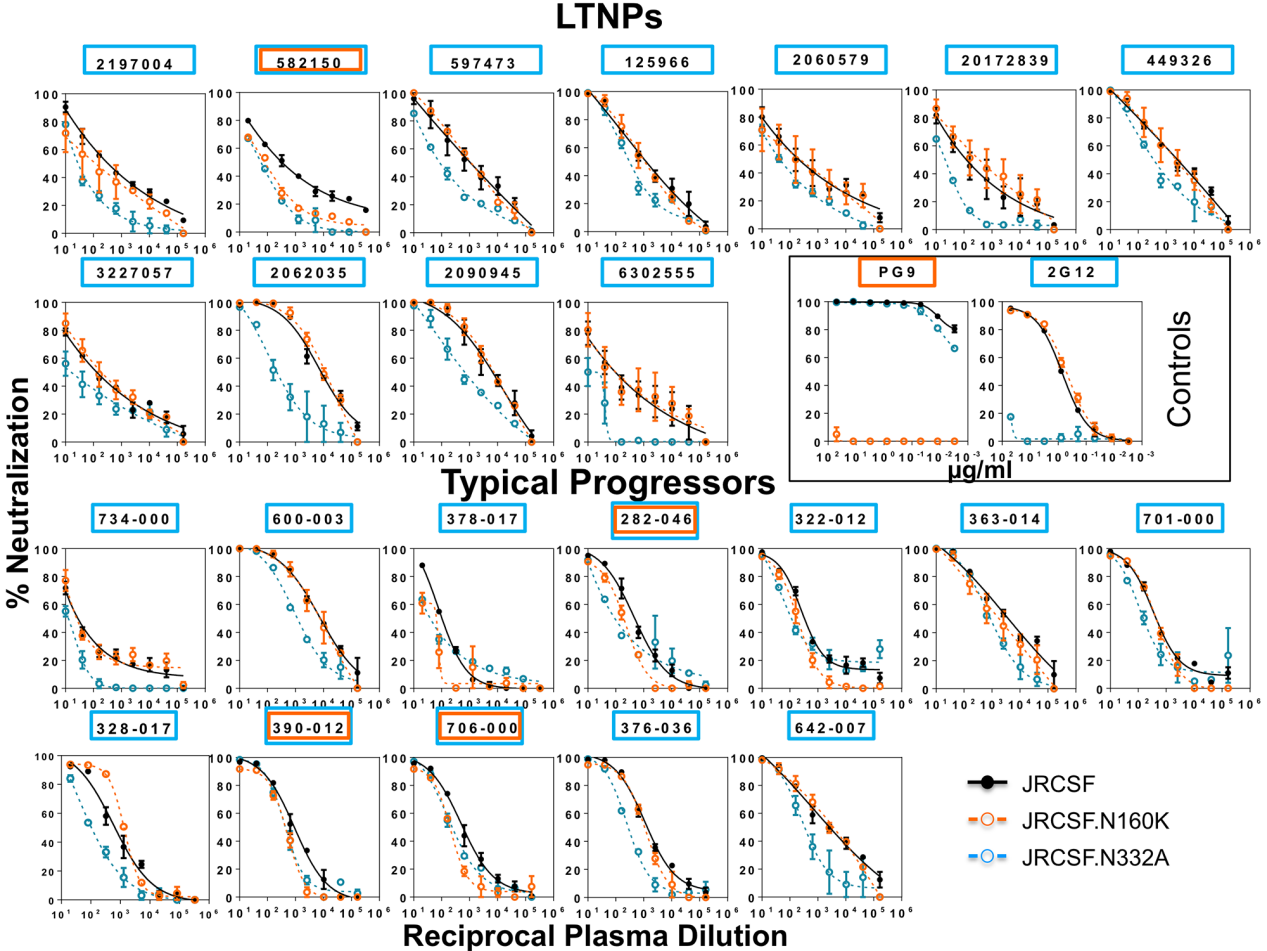
Detection of glycan-dependent HIV-1 neutralizing antibodies in sera from LTNPs and typical progressors. Sera with V1V2 and V3 glycan-dependent HIV-1 NAbs are detected by neutralization assays with the parental JRCSF and the corresponding N160K and N332A mutants (V1V2 and V3 respectively). Sera with reactivity to glycan-dependent motives in V2 and V3 are indicated in orange and blue respectively. Percent serum neutralizing activity sensitive to the indicated residue replacement was calculated using the equation [1 − (ID50 mutant/ID50 wild type)] x 100. A sample is considered positive if there is a decrease in ID50 greater than or equal to 50% for the mutant relative to the wild-type virus. Monoclonal antibodies 2G12 and PG9 have been used as controls and are indicated by a black box. SEMs of two independent assays are shown. In this figure only samples with neutralizing antibodies directed to glycans in V1V2 and/or V3 are shown.

For the mapping of V1V2 and V3 glycan-dependent HIV-1 NAbs (Fig 3 and S2 Fig), neutralization assays in TZM-bl using JRCSF.N160K and JRCSF.N332A respectively were performed. A decrease of 2 fold or more in serum neutralization against JRCSF.N160K or JRCSF.N332A compared to the neutralization of wild-type JRCSF indicates the presence of neutralizing antibodies directed to glycans in V1V2 or V3, respectively. From the results obtained it can be deduced that glycan-dependent HIV-1 NAbs are significantly more abundant in the samples from LTNPs (11/12) than in those from TPs (12/33) (p value = 0.0017, Fisher’s exact test).

**Fig 4:**
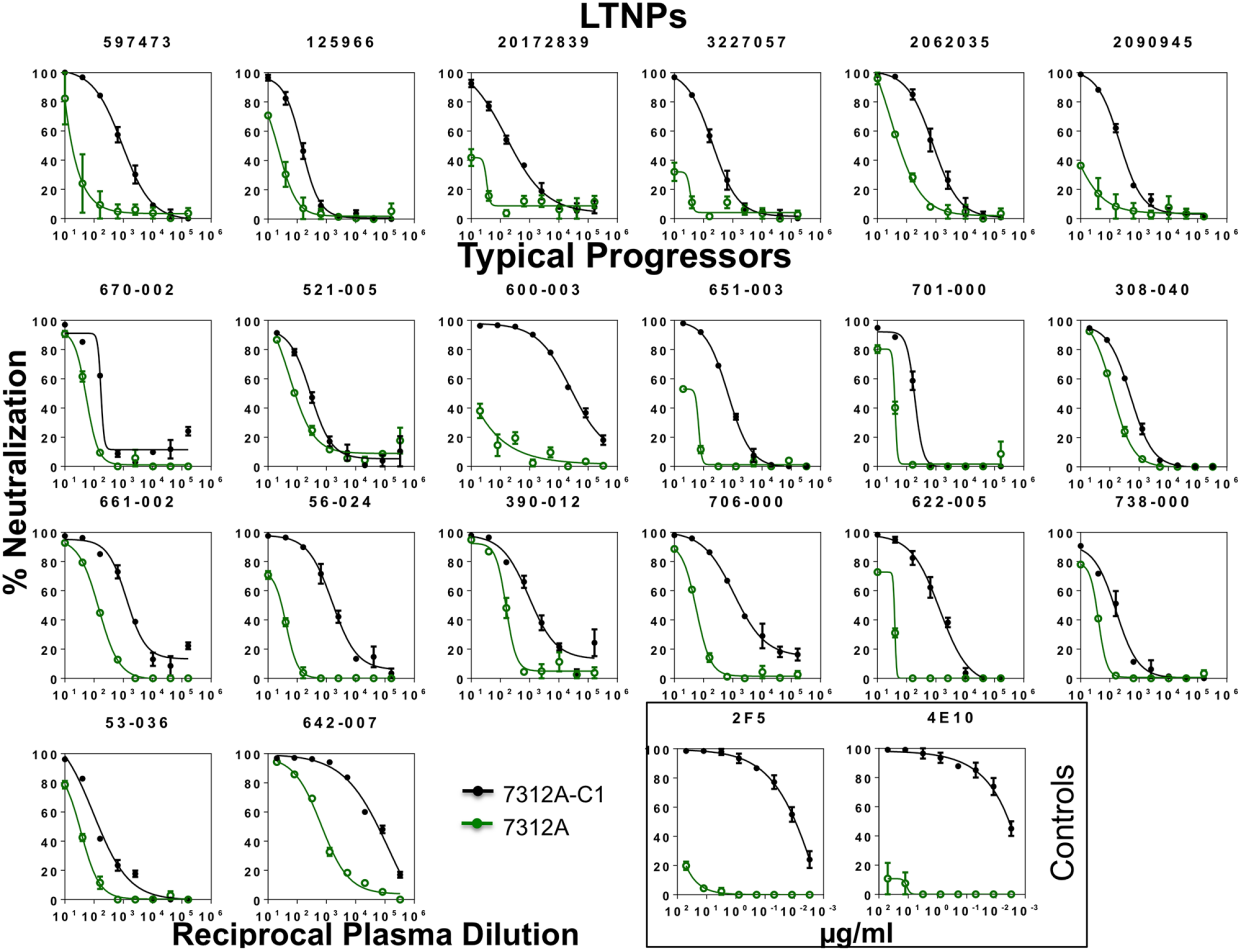
Detection of antibodies specific for the membrane-proximal region in the sera from LTNPs and typical progressors. For the mapping of anti-MPER neutralizing antibodies, the serum samples were tested against the parental HIV-2 isolate 7312A and the 7312A chimera containing HIV-1 MPER fragments (7312A-C1). Monoclonal antibodies 2F5 and 4E10 have been used as controls and are indicated by a black box. In this figure only samples with anti-MPER neutralizing antibodies are shown.

Sera were analyzed to detect antibodies directed against MPER region of gp41 (Fig 4 and S3 Fig) assessing neutralizing activity against 7312A and 7312A-C1 viruses and anti-MPER antibodies were detected in both groups of patients. To confirm the presence of MPER neutralizing antibodies in the sera, the specificity of the antibodies against MPER was inhibited with soluble peptides containing different fragments of this region (Table 4). Four serum samples from LTNPs and eight serum samples from TPs competed with the MPR.03 peptide that covers the complete MPER domain. One of the LTNP samples (2090945) contained antibodies similar to 4E10. One serum sample from TPs (53–036) contained bNAbs directed against the same epitope than Z13 and five TP samples (600–003, 701–000, 390–012, 622–005 and 642–007) contained bNAbs directed against the same epitope than 4E10. The epitope of one TP sample (56–024) overlapped that of both 4E10 and 2F5 and the epitope of other TP sample (661–002) overlapped that of 4E10 and Z13e1.

**Table 4:** ID50 titers in the absence and the presence of MPER-derived peptides.

Serum	ID50						% Neutralization inhibited by peptide*				
	Mock	MPR.Scr.02	MPR.03	2F5.01	4E10.22	z13e1.01	MPR.Scr.02	MPR.03	2F5.01	4E10.22	z13e1.01
**LTNPs**											
**ECs**											
597473	580	541	365	533	711	423	7	37	8	0	27
125966	85	100	92	89	117	103	0	0	0	0	0
20172839	241	126	37	210	128	111	48	**84**	13	47	**54**
**VCs**											
3227057	129	123	61	98	113	91	5	**52**	24	12	29
2062035	357	425	162	511	380	470	0	**55**	0	0	0
2090945	111	134	27	90	38	118	0	**75**	19	**66**	0
**TPs**											
600–003	4047	5444	12	5823	10	2563	0	**100**	0	**100**	37
651–003	797	371	345	548	647	327	**53**	**57**	31	19	**59**
701–000	2779	1521	227	1284	677	1447	45	**92**	**54**	**76**	48
661–002	2244	2236	771	1730	973	853	0	**66**	23	**57**	**62**
56–024	3279	3216	94	1336	146	7461	2	**97**	**59**	**96**	0
390–012	1494	1306	712	1806	591	856	13	**52**	0	**60**	43
706–000	1539	896	2313	876	993	814	42	0	43	35	47
622–005	773	1014	170	407	82	390	0	**78**	47	**89**	50
53–036	164	121	46	119	135	46	26	**72**	27	18	**72**
642–007	5932	8449	588	8646	375	4900	0	**90**	0	**94**	17

*Indicator of peptide-specific neutralizing antibody response, calculated as (1 –ID50 with peptide/ID50 with mock peptide) x 100. Values higher than 50% are shown in bold

**Fig 5:**
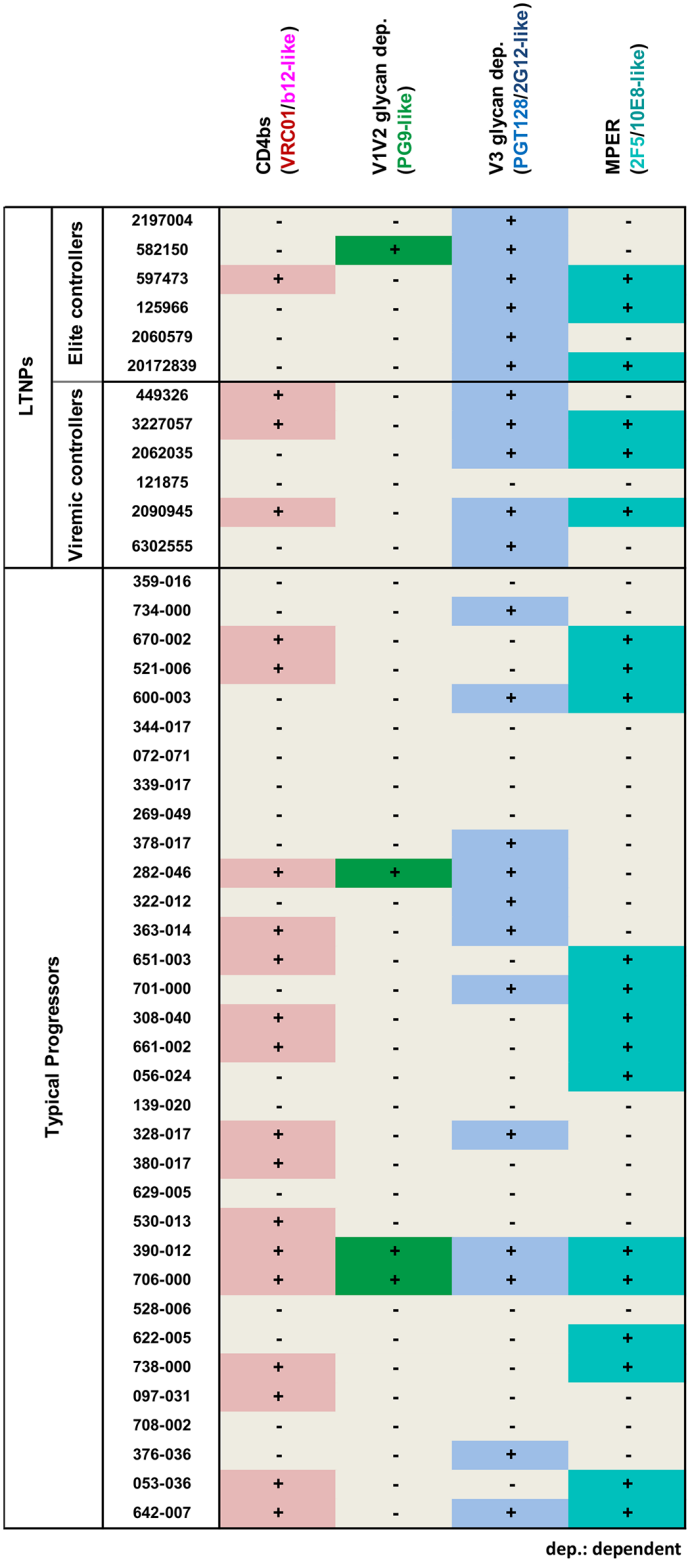
Summary of experimental serum mapping of HIV-1 sera from LTNPs and TPs with a broad neutralization profile. Data obtained from the assays used to map CD4bs, V1V2 glycans, V3 glycans and MPER regions are shown. Epitope groups are marked with a plus sign (+) if predicted by the mapping assays to be present in a given serum.

**Fig 6:**
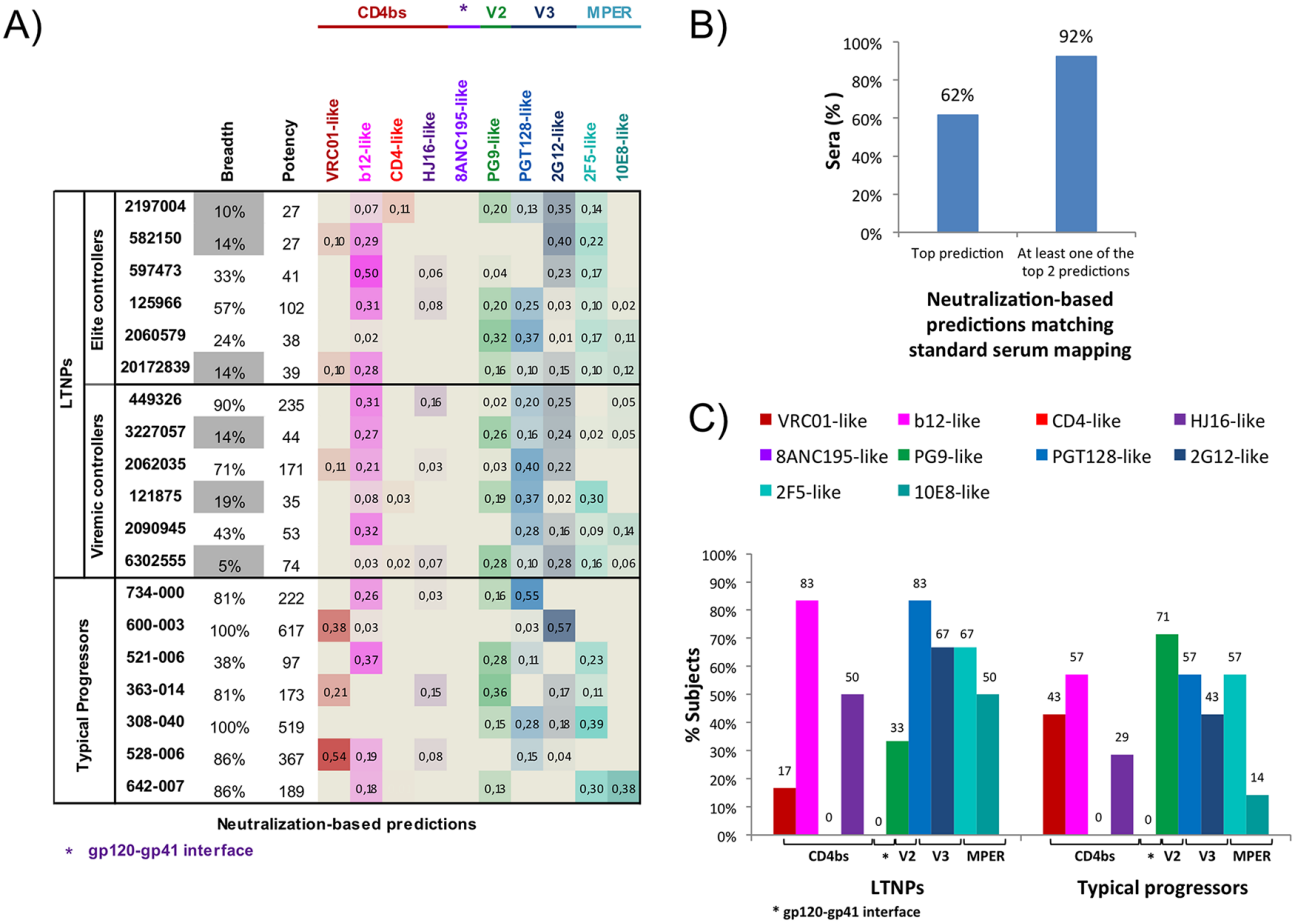
Delineation of serum specificities in HIV-1 sera from LTNPs and TPs with a broad neutralization profile. **(A)** Predictions based on the serum neutralization pattern against a panel of diverse HIV-1 isolates are shown on the left of the figure. For each serum the predicted relative prevalence of each reference antibody cluster is presented as a heat map where stronger neutralization signals by the antibody cluster are shown as darker colors (higher fractional numbers). Breadth was measured as the percentage of viruses neutralized with an ID50>50 and potency as the geometric mean ID50 titers of sera. Sera with breadth less than 20% are greyed out since the computational predictions in those cases are less reliable **(B)** Concordance between neutralization-based and standard serum mapping. Sera with breadth less than 20% were excluded. The agreement between the two methods was good. In 92% of the sera at least one of top two computational neutralization-based predictions was identified by standard mapping. **(C)** Frequency of the epitope specificities of HIV-1 neutralizing antibodies in the sera from LTNPs and TPs with broadly neutralizing activity. Sera with breadth less than 20% were excluded.

In summary, we found that most of the bNAbs from LTNPs map known neutralization epitopes and that in some subjects the neutralization breadth is mediated by antibodies with different specificities (Figs 5 and 6). A summary of serum neutralization specificities found with standard mapping is shown (Fig 5).

It has been shown that similarity in neutralization fingerprint correlates with similarity in epitope [42]. Therefore, epitope specificities of HIV-1–neutralizing antibodies in serum were elucidated from the serum pattern of neutralization against a panel of 21 HIV-1 isolates. The patterns of neutralization of the sera were compared with a reference set of 10 epitope-specific neutralization fingerprints (one for each epitope-specific antibody cluster). The predicted prevalence of the different clusters is shown as a heat map for each serum (Fig 6A). These data exhibited a high concordance with the ones obtained using the experimental assays (Fig 5), with at least one of the top two neutralization-based specificities identified by standard mapping in 92% of the sera (Fig 6B). Epitopes involved in bNAbs-mediated protection have been characterized by these different approaches and activity against all the analyzed epitopes was displayed in LTNPs and TPs (Fig 6C).

## Discussion

It has been suggested that in HIV-infected individuals high levels of viral replication and the time since infection correlates with the induction of bNAbs [1,21,46]. However, in this work we have detected broadly neutralizing antibodies against HIV-1 in a cohort of LTNPs with low or undetectable levels of viremia. Actually, LTNP were classified in two sub-groups according to viremia levels: elite controllers (persistent undetectable viremia) and viremic controllers (VL<10.000 copies/ml). Although we have found a higher percentage of individuals with bNAbs in viremic controllers (14% vs 7%), bNAbs were also found in long-term elite controllers, suggesting that other factors besides persistently detected viremia could drive the development of bNAbs. However, we cannot exclude that this broad humoral immune response could be due to hidden viral replication in other tissues such as gut-associated lymphoid tissue (GALT) or tonsils.

LTNPs and TPs developed antibodies against all kinds of epitopes analyzed. Therefore, bNAbs in LTNPs are mapped to specific known neutralization epitopes. These data have been obtained using two different approaches, neutralization-based and standard serum mapping. The concordance between these two methods was high. These two approaches have been previously compared in a population of 21 sera [42]. The data obtained in the present study of 19 additional sera validate the previous reports of concordance between neutralization fingerprinting and standard serum mapping, further underlining the utility of the neutralization-based serum-epitope predictions.

Only two of the sera with binding antibodies against the CD4bs detected by ELISA had detectable neutralization activity confirmed by RSC3 competition neutralization assays. One possible explanation is that if the virus is neutralized by antibodies directed against other epitope, there will not be an effect on neutralization reduction mediated by RSC3. The sera samples with no inhibition in neutralization by this glycoprotein could have few antibodies against CD4bs or antibodies with low affinity incapable of mediating neutralization, only detectable by ELISA techniques. Then, in these sera the neutralization may be due mainly to the presence of high-affinity antibodies against other domains.

When we analyzed the sera samples with the standard serum mapping, we observed that V3 glycan-dependent HIV-1 NAbs were more abundant in LTNPs (11/12) than in TPs (12/33). This prevalence of the V3 glycan-dependent NAbs in LTNPs was also detected with the neutralization-based analysis. A previous study has also found high levels of 2G12-like antibodies in broadly neutralizing samples from LTNPs [47]. A question that arises from these results is whether these antibodies are contributing to the control of viremia in LTNP. The V3 region is known to be highly immunogenic and individuals develop antibodies directed against the C3-V4 region early in infection [48,49]. HIV overcomes the response through mutation but this variability is decreased in the low viral rates of replication. It could be possible that in some LTNPs bNAbs contribute to viral control. Actually it has been recently described that Abs from one EC patient can exhibit autologous neutralization and these antibodies are contributing to elite control in this individual [22]. To address this hypothesis autologous neutralization should be detected in LTNP patients but due to extremely low viral loads, viral isolation and cloning of the envelope is unfeasible in the majority of patients.

In some subjects neutralization breadth was mediated by more than one antibody epitope specificity which is in agreement with previous observations showing that broadly neutralizing activity of some HIV-1 infected individuals is due to antibodies that target more than one epitope [38,50–52].

bNAbs require a large number of somatic mutations [7] that are related with preservation of T follicular helper cells (TFH). HIV infected individuals have several defects in the humoral immune system, including B cell abnormalities associated with HIV replication-induced immune cell activation and TFH priming [53–55]. Elite controllers with viral load consistently below 50 copies/ml could develop a robust response mediated by their well-preserved B cells generating high affinity antibodies. Therefore control of the viral load could be associated to an improved maturation of antibodies in the affinity for the antigen. TFH are involved in the development of these antibodies as B cell memory maturation and generation of high-affinity neutralizing antibodies is dependent on extensive signaling from TFH cells [56]. However, in productive HIV infection, high levels of HIV viremia drive the expansion of TFH cells which is associated with perturbation of the B cell compartment, resulting in deregulated antibody production [57,58]. One potential hypothesis could point to a better preserved B cell function in LTNPs including appropriate regulation of TFH resulting in a generation of bNAbs with high levels of somatic hypermutation despite lower levels of antigen.

## Supporting information

S1 Fig Neutralizing activity of sera from LTNPs and TPs. Percentages of neutralization at a 1/200 serum dilution against the mini-panel of viruses (NL4-3, VI191, 92BR025 and CM244). A white box indicates <50% neutralization, a yellow box indicates ≥50% and <70% neutralization, an orange box indicates ≥70% and <90%and a red box indicates ≥90% neutralization.
(PPTX)

S2 Fig Detection of glycan-dependent HIV-1 neutralizing antibodies in sera from LTNPs and typical progressors (negative samples). In this figure only samples with no neutralizing antibodies directed to glycans in V1V2 and/or V3 are shown. SEMs of two independent assays are shown.
(PPTX)

S3 Fig Detection of antibodies specific for the membrane-proximal region in the sera from LTNPs and typical progressors (negative samples). In this figure only samples with no neutralizing antibodies specific for the membrane-proximal region are shown. SEMs of two independent assays are shown.
(PPTX)

S4 Fig Serum neutralization data (ID50s) for LTNPs and TPs against the panel of 21 HIV-1 isolates used in the antibody-sera delineation analysis. Reciprocal serum ID50 values ≥40 and <500 are highlighted in yellow, ≥500 and <5000 in orange and ≥5000 in red. For VRC01 IC50 values ≥1 and <10 are highlighted in yellow, ≥0.100 and <1 in orange and <0.100 in red.
(PPTX)

S1 Table Serum neutralization activity (ID50) against JRFL, RW020 and ZA012 viruses used in RSC3 neutralization competition assays. (DOCX)
