# Supplementary figures and images for: Characterization of broadly neutralizing antibody responses to HIV-1 in a cohort of long term non-progressors
Source: PLoS One. 2018 Mar 20;13(3):e0193773. doi: 10.1371/journal.pone.0193773 (PMC5860703; doi:10.1371/journal.pone.0193773)

## Slide 1
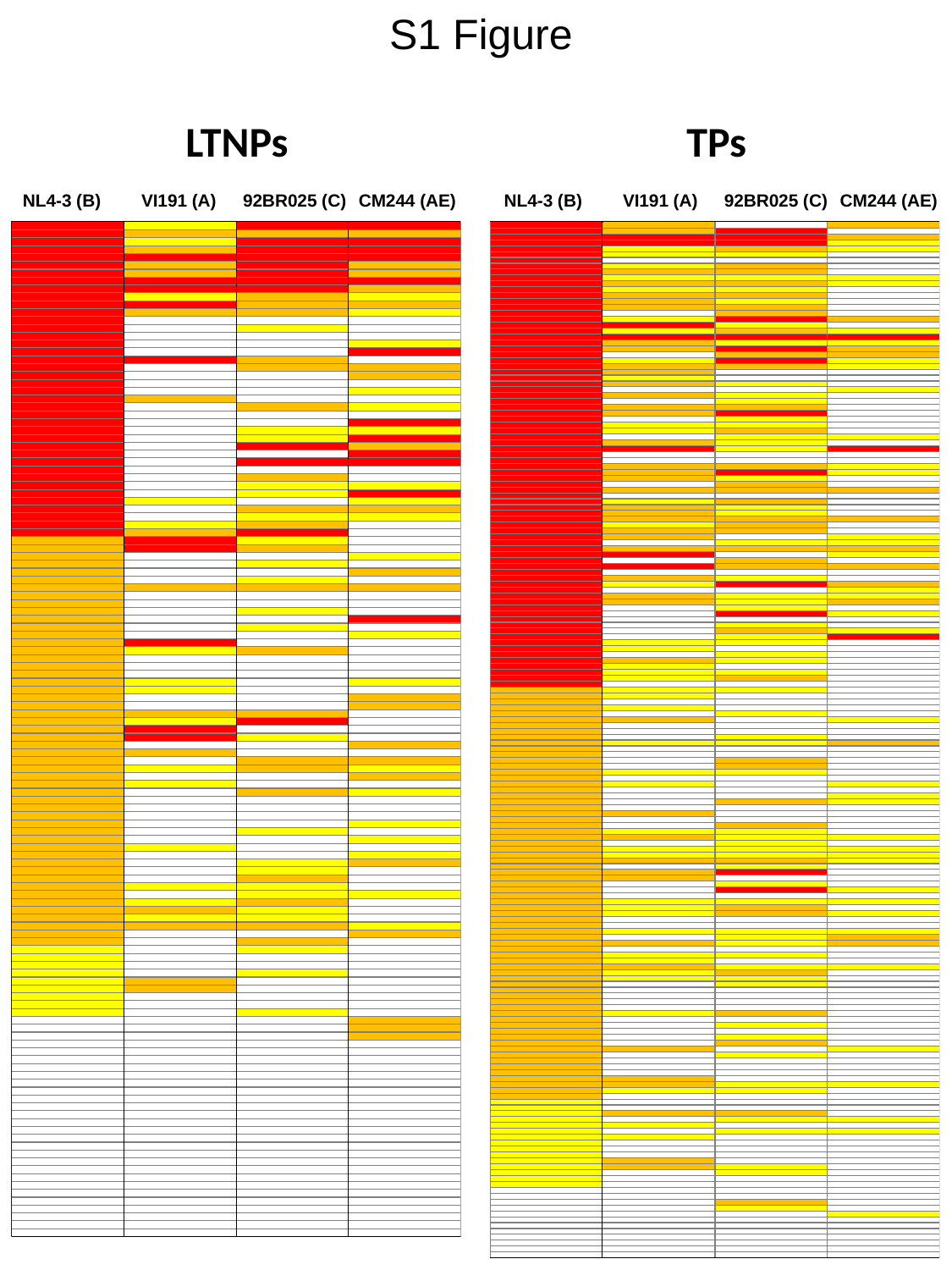

S1 Figure
LTNPs
TPs
NL4-3 (B)
VI191 (A)
92BR025 (C)
CM244 (AE)
NL4-3 (B)
VI191 (A)
92BR025 (C)
CM244 (AE)

Supplement: S1 Fig — Percentages of neutralization at a 1/200 serum dilution against the mini-panel of viruses (NL4-3, VI191, 92BR025 and CM244). A white box indicates <50% neutralization, a yellow box indicates ≥50% and <70% neutralization, an orange box indicates ≥70% and <90%and a red box indicates ≥90% neutralization. (PPTX) [file pone.0193773.s001.pptx]

## Slide 1
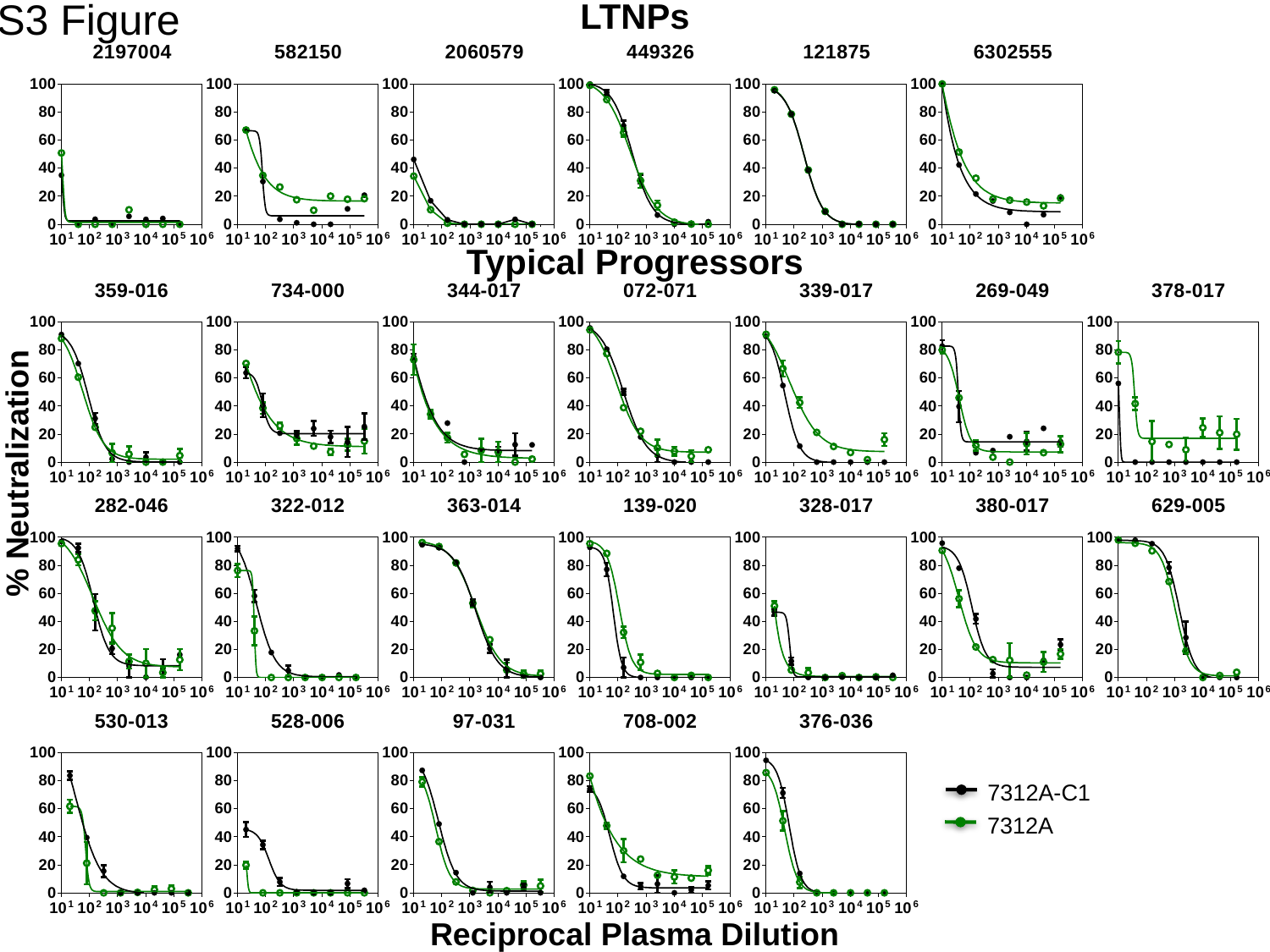

S3 Figure
LTNPs
Typical Progressors
% Neutralization
7312A-C1
7312A
Reciprocal Plasma Dilution

Supplement: S3 Fig — In this figure only samples with no neutralizing antibodies specific for the membrane-proximal region are shown. SEMs of two independent assays are shown. (PPTX) [file pone.0193773.s003.pptx]

## Slide 1
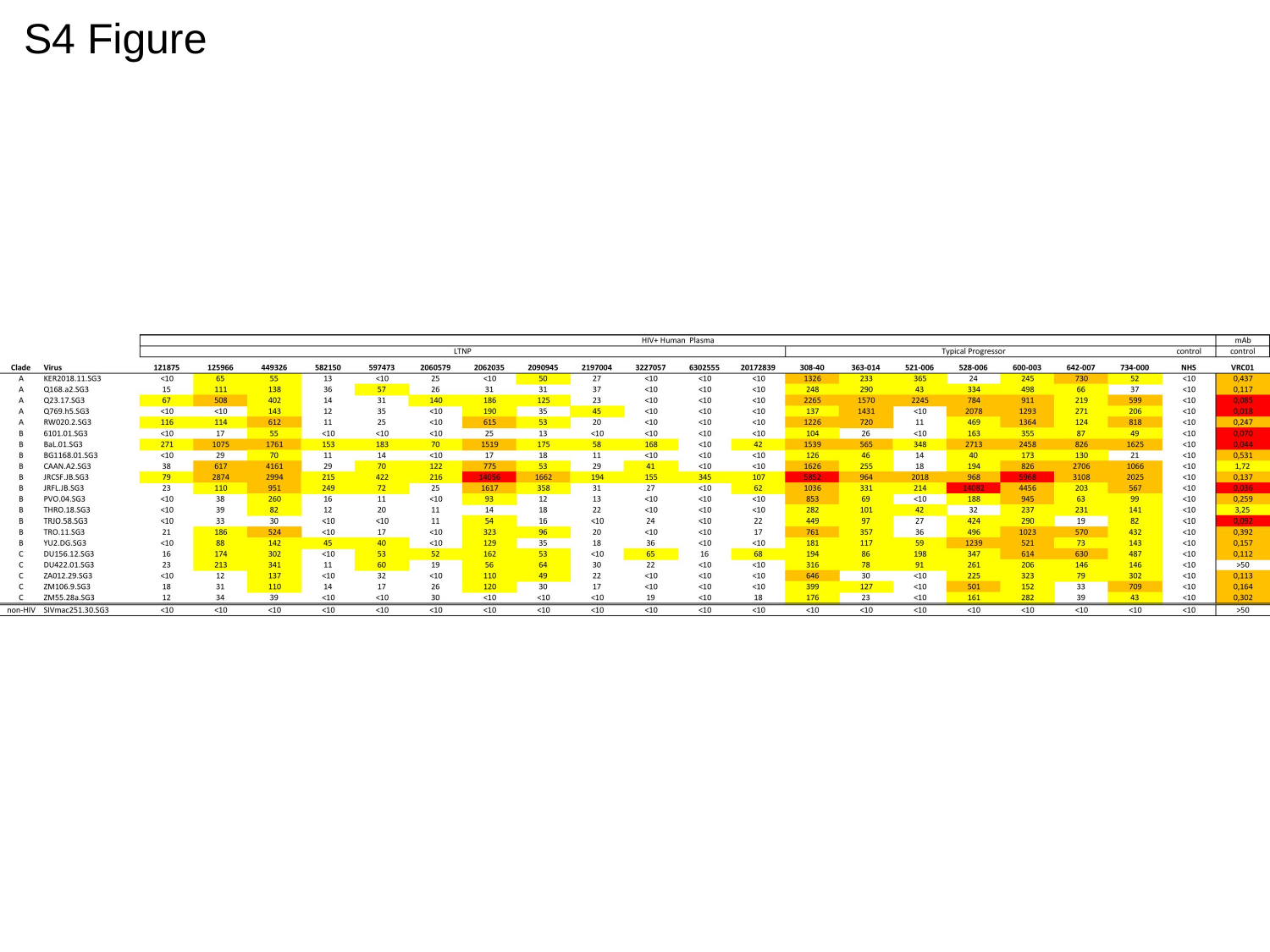

S4 Figure

Supplement: S4 Fig — Reciprocal serum ID50 values ≥40 and <500 are highlighted in yellow, ≥500 and <5000 in orange and ≥5000 in red. For VRC01 IC50 values ≥1 and <10 are highlighted in yellow, ≥0.100 and <1 in orange and <0.100 in red. (PPTX) [file pone.0193773.s004.pptx]
