# Supplementary material for: Characterization of broadly neutralizing antibody responses to HIV-1 in a cohort of long term non-progressors
Source: PLoS One. 2018 Mar 20;13(3):e0193773. doi: 10.1371/journal.pone.0193773 (PMC5860703; doi:10.1371/journal.pone.0193773)
Supplement: S2 Fig — In this figure only samples with no neutralizing antibodies directed to glycans in V1V2 and/or V3 are shown. SEMs of two independent assays are shown. (PPTX) [file pone.0193773.s002.pptx]

## Slide 1
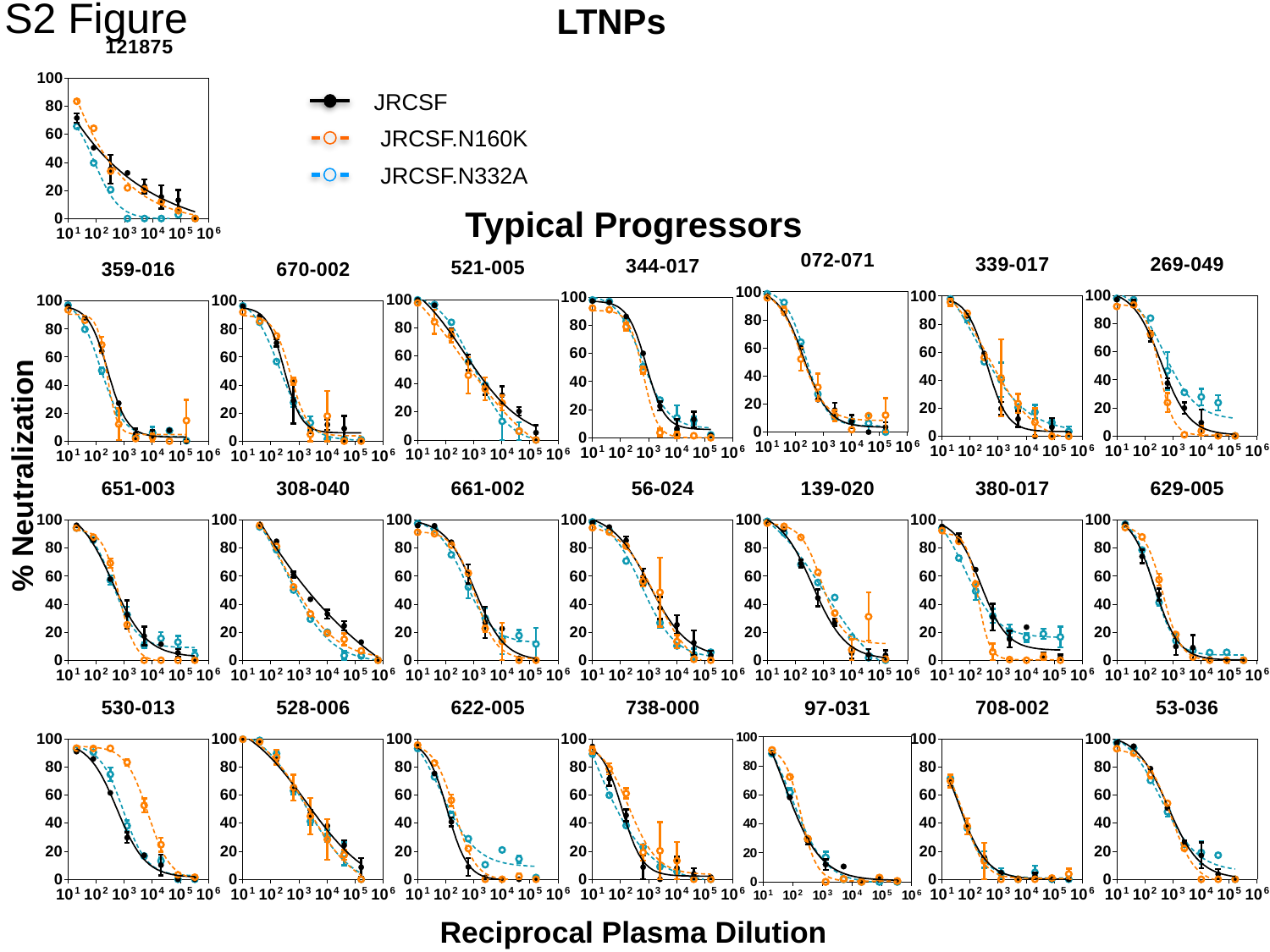

S2 Figure
LTNPs
JRCSF
JRCSF.N160K
JRCSF.N332A
Typical Progressors
% Neutralization
Reciprocal Plasma Dilution
