# Supplementary material for: Characterization of broadly neutralizing antibody responses to HIV-1 in a cohort of long term non-progressors
Source: PLoS One. 2018 Mar 20;13(3):e0193773. doi: 10.1371/journal.pone.0193773 (PMC5860703; doi:10.1371/journal.pone.0193773)
Supplement: S1 Table — (DOCX) [file pone.0193773.s005.docx]

**S1 Table. Serum neutralization activity (ID50) against JRFL, RW020 and ZA012 viruses used in RSC3 neutralization competition assays**

|  | **JRFL** | **RW020** | **ZA012** |
| --- | --- | --- | --- |
| **449326** | 550 | 680 | 43 |
| **597473** | 91 | 26 | 81 |
| **2090945** | 344 | 53 | 44 |
| **3227057** | 68 | 28 | 10 |
| **308-40** | 455 | 363 | 178 |
| **363-014** | 450 | 81 | 73 |
| **521-06** | 232 | 80 | 93 |
| **642-07** | 362 | 128 | 122 |
